# Impact of the creation of a multidisciplinary amyloidosis study group in a public hospital of a developing Latin American country

**DOI:** 10.1016/j.htct.2025.103820

**Published:** 2025-04-12

**Authors:** Camila Peña, José Manuel Matamala, Cristián Vargas, Jaime Álvarez, Ricardo Valjalo, Fernando J. Verdugo

**Affiliations:** a Hematology Unit, Hospital del Salvador, Santiago, Chile; b Department of Internal Medicine, Faculty of Medicine, University of Chile, Santiago, Chile; c Center for Advance Clinical Research (CICA) Oriente, Faculty of Medicine, University of Chile, Santiago, Chile; d Translational Neurology and Neurophysiology Laboratory (NODO Lab), Faculty of Medicine, University of Chile, Santiago, Chile; e Department of Neurological Sciences, Faculty of Medicine, University of Chile, Santiago, Chile; f Biomedical Neuroscience Institute (BNI), Faculty of Medicine, University of Chile, Santiago, Chile; g Internal Medicine Service, Hospital del Salvador, Santiago Chile; h Cardiology Unit, Hospital del Salvador, Santiago, Chile; i Nephrology Unit, Hospital del Salvador, Santiago, Chile

**To the editor:**

Amyloidosis represents a diagnostic challenge due to low clinical suspicion and the technical difficulties involved in correctly typing amyloid. Recognition of the clinical picture is usually delayed, in part due to its multisystemic and nonspecific involvement. Patients may spend between seven months and more than five years visiting various specialists and receiving multiple incorrect diagnoses before obtaining the correct diagnosis.^1^ Moreover, most treatments are expensive, which makes it a difficult disease to manage in developing countries.

In 2015 an alliance between cardiology and hematology specialists was made at our center in order to evaluate patients with suspected amyloidosis. In 2017, an amyloidosis study group was formally created, made up of specialists in hematology, cardiology, nephrology, neurology, dermatology, and pathology.

The aim of this study was to evaluate the impact of the creation of an Amyloidosis Multidisciplinary Study Group in a public center of a developing Latin American country.

An observational analytic ambispective cohort study was made. This study included all patients in the amyloidosis registry of our center (Hospital del Salvador, Santiago de Chile) diagnosed between 2005 and 2022. We divided the cohort into two groups - Period 1 (P1): patients diagnosed from 2005 to 2014 (before the Amyloidosis Study Group), and Period 2 (P2) from 2015 to 2022 (after establishing the Amyloidosis Study Group). Comparisons between the groups were performed using the *t*-test or Chi-square test. Overall survival (OS) was estimated using Kaplan Meier curves and comparisons were made by the Log Rank test. The analyzes were performed using the Statistical Package for Social Sciences (SPSS) computer program version 26.0. The study was approved by the local Ethics Committee.

Fifty-six patients with diagnosis of amyloidosis were included: 12 in P1 and 44 in P2 (Figure 1). The median ages were 63 and 66 years-old (p-value = 0.38) in P1 and P2, respectively and 67% versus 39% were male (p-value = 0.08). All cases were amyloid light-chain amyloidosis (i.e. primary amyloidosis - AL) in P1, while in P2 there were also two cases of secondary amyloidosis (AA) and two cases of hereditary transthyretin amyloidosis (ATTRm).

**Figure 1:**
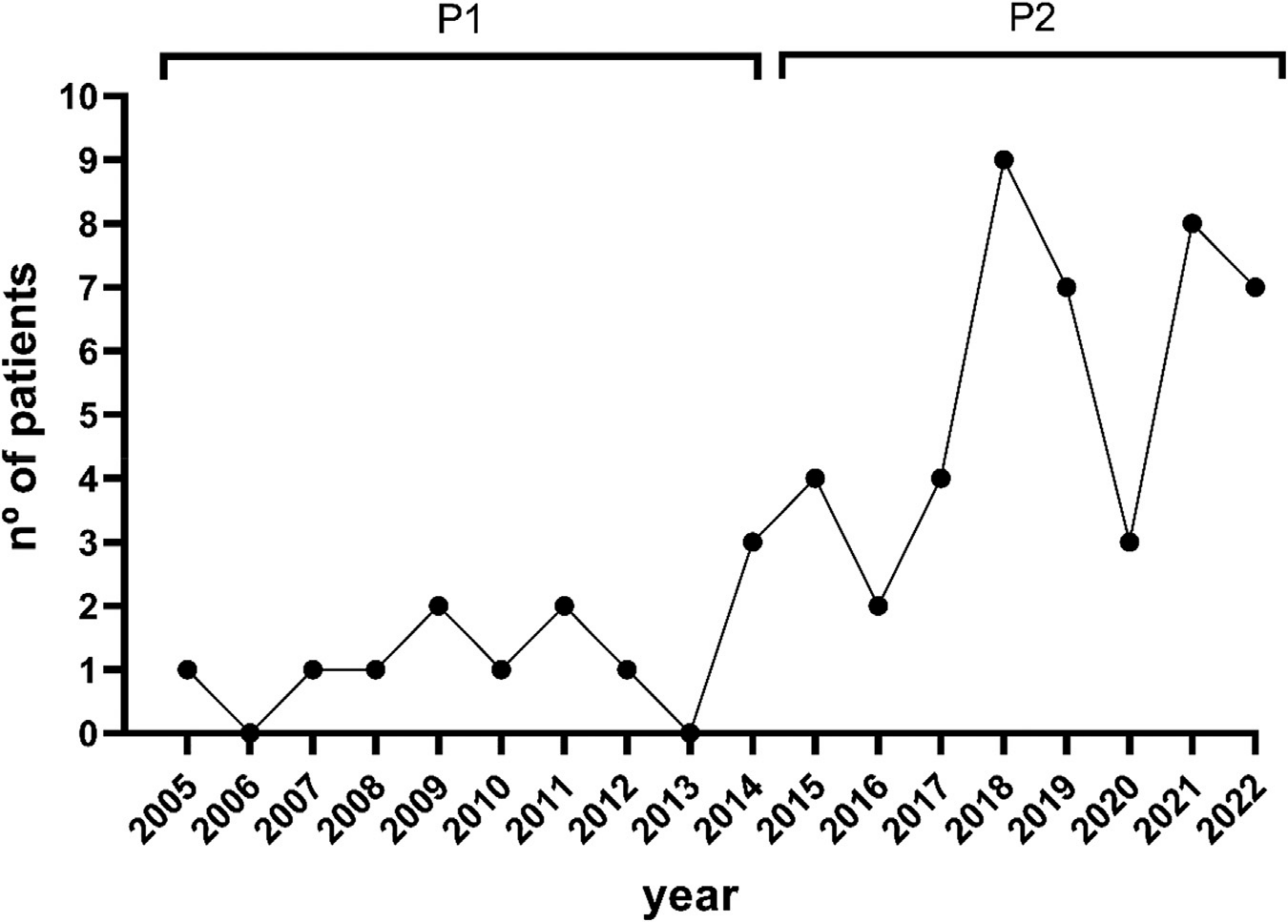
Number of patients diagnosed with amyloidosis per year.

Analysis in regard to the availability of diagnostic and prognostic tools between P1 and P2, respectively was as follows: echocardiography was performed in 58% versus 93% of the patients (p-value < 0.001), the longitudinal strain was estimated in echocardiograms in 0% versus 61% (p-value < 0.001), cardiac magnetic resonance imaging (MRI) was performed in 0% versus 14% (p-value = 0.176), N-terminal pro-B-type natriuretic peptide (NT-proBNP) was evaluated in 8% versus 68% (p-value < 0.001), and the free light chain assay was performed in 25% versus 82% of the cases (p-value < 0.001).

No treatment based on bortezomib was prescribed in P1, and most patients were treated with a melphalan-prednisone regimen. In P2, 45% of patients were induced with a bortezomib (Bortezomib)-based regimen (p-value = 0.004). The early mortality rate was 67% in P1 and 30% in P2 (p-value = 0.020). The estimated five-year OS of the cohort in P1 was 16.7% versus 43.6% in P2 (p-value = 0.017 - Figure 2).

**Figure 2:**
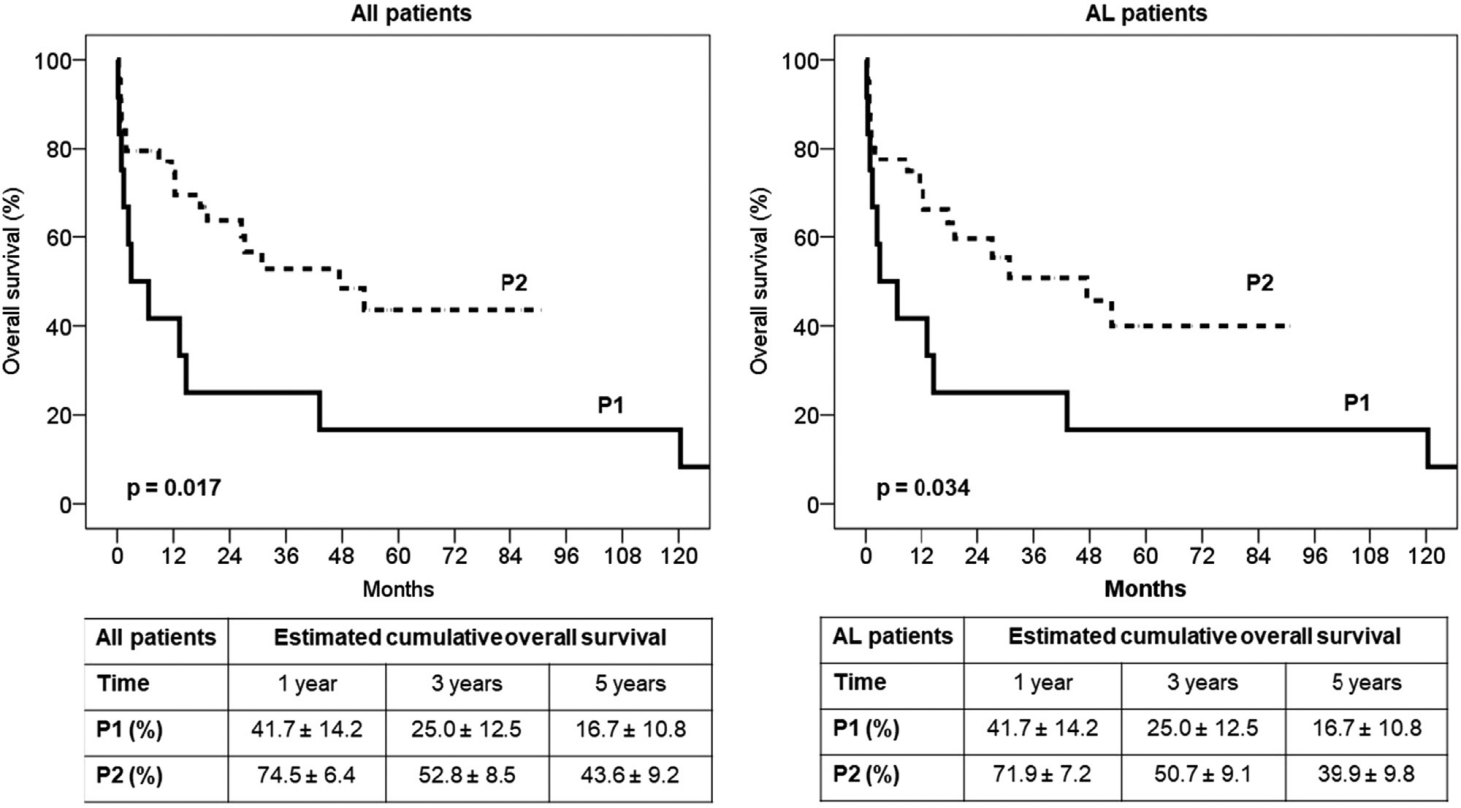
Overall survival for the periods 2005–2014 (P1) and 2015–2022 (P2) for the whole cohort and for AL patients.

Since the creation of the group, the diagnosis of amyloidosis clearly improved with a better access to diagnostic and prognostic tools.

Amyloidosis is considered an orphan disease, which is chronically debilitating, serious, and life-threatening. Because of this, it must be addressed in a particular way. Worldwide, several measures have been proposed in this regard including: education, support for research, the possibility of entering in clinical trials, requesting equitable access to appropriate diagnosis and treatment, and the creation of multidisciplinary teams for its study. Our results prove that better management of these patients can be achieved, without necessarily meaning a large increase in the budget.

This group has focused mainly on constant education, both for specialists and non-specialist physicians. We have also managed to incorporate basic tests, such as echocardiography with longitudinal strain estimation, free light chain assays, and measurement of NT-ProBNP and troponin levels. More recently, access to cardiac MRI was added for selected patients. Moreover, since 2018 we can treat AL amyloidosis with bortezomib-based induction.

The most important result is that the diagnosis of amyloidosis improved progressively, except in 2020, which can be explained by the shutdown during the COVID-19 pandemic. This possibly means there has been an increased awareness of the disease in our center.

We were also able to diagnose other types of amyloidosis: two cases of AA and two of ATTRm. The latter were the first patients with ATTRm with a neurological phenotype diagnosed in our country.^2^ This milestone was achieved thanks to the incorporation of immunohistochemistry for AA and genetic testing for suspected ATTRm. In P2, there was greater access to all relevant diagnostic and prognostic tools, although unfortunately this was not for all patients. This will improve in coming years, when we will have greater availability of these tests, including cardiac MRI. Wild-type transthyretin amyloidosis (ATTRwt) remains undiagnosed. In the future, one of the goals of our group is to incorporate pyrophosphate scintigraphy to our diagnostic arsenal according to the currently recommended non-invasive diagnostic algorithm.^3^

In regard to treatment, we experienced great improvements as, in 2018 we incorporated bortezomib to our treatment arsenal, as previously mentioned. We hope we soon have access to the anti-CD38 monoclonal antibody daratumumab, as daratumumab-CyBorD became standard of care for AL amyloidosis.^4^ Access to disease-modifying therapy for ATTR has been restricted due to high costs and the absence of a national funding policy. Nevertheless, we recently started tafamidis treatment in an ATTRm patient with late-onset cardiovascular involvement.

We observed a relevant decrease in the early mortality rate and a better OS in P2, which we believe reflects the joint efforts with increased awareness, early diagnosis, and prompt treatment using improved therapeutic drugs.

Our study has several limitations, including its ambispective and unicentric nature, and the relatively low number of patients included. Nevertheless, it seems relevant to report that an improvement in both diagnosis and treatment is possible, even in poorer countries.

We are aware that there is still a long way to go to reach international standards. Our next step will be to start performing microdissection and mass spectrometry in biopsies for a better characterization, cardiac MRI and technetium-99 m pyrophosphate scintigraphy imaging, with the final goal of becoming a national reference center.

## Funding

This research did not receive any specific grant from funding agencies in the public, commercial, or not-for-profit sectors.

## Conflicts of interest

The authors declare no conflicts of interest.
